# Design, preparation, and combustion performance of energetic catalysts based on transition metal ions (Cu^2+^, Co^2+^, Fe^2+^) and 3-aminofurazan-4-carboxylic acid†

**DOI:** 10.1039/d3ra03585a

**Published:** 2023-09-05

**Authors:** Wei Liu, Yuangang Xu, Yulong Zhang, Hanyue Zheng, Xiaodong Gou, Fei Xiao

**Affiliations:** a School of Environmental and Safety Engineering, North University of China Taiyuan Shanxi China 1104054142@st.nuc.edu.cn; b School of Chemistry and Chemical Engineering, Nanjing University of Science and Technology Nanjing Jiangsu China yuangangxu@163.com; c China North Industry Advanced Technology Generalization Institute Beijing China

## Abstract

Development of energetic catalysts with high energy density and strong catalytic activity has become the focus and frontier of research, which is expected to improve the combustion performance and ballistic properties of solid propellants. In this work, three energetic catalysts, M(H_2_O)_4_(AFCA)_2_·H_2_O (AFCA = 3-aminofurazan-4-carboxylic acid, M = Cu, Co, Fe), are designed and synthesized based on the coordination reaction of transition metal ions and the energetic ligand. The target products are characterized by single crystal X-ray diffraction, Fourier transform infrared spectroscopy, differential thermal analysis, optical microscopy, and scanning electron microscopy. The results reveal that Cu(H_2_O)_4_(AFCA)_2_·H_2_O crystallizes in the monoclinic space group, *D*_c_ = 1.918 g cm^−3^. Co(H_2_O)_4_(AFCA)_2_·H_2_O, and Fe(H_2_O)_4_(AFCA)_2_·H_2_O belong to orthorhombic space groups, their density is 1.886 g cm^−3^ and 1.856 g cm^−3^, respectively. In addition, the designed catalysts show higher catalytic activity than some reported catalysts such as Co(en)(H_2_BTI)_2_]_2_·en (H_3_BTI = 4,5-bis(1*H*-tetrazol-5-yl)-1*H*-imida-zole), Co-AzT (H_2_AzT = 5,5′-azotetrazole-1,1′-diol), and [Pb(BTF)(H_2_O)_2_]_*n*_ (BTF = 4,4′-oxybis [3,3′-(1-hydroxy-tetrazolyl)]furazan) for the thermal decomposition of ammonium perchlorate (AP). The high-temperature decomposition peak temperatures of AP/Cu(H_2_O)_4_(AFCA)_2_·H_2_O, AP/Co(H_2_O)_4_(AFCA)_2_·H_2_O, and AP/Fe(H_2_O)_4_ (AFCA)_2_·H_2_O are decreased by 120.3 °C, 151.8 °C and 89.5 °C compared to the case of pure AP, while the heat release of them are increased by 768.8 J g^−1^, 780.5 J g^−1^, 750.9 J g^−1^, respectively. Moreover, the burning rates of solid propellants composed of AP/Cu(AFCA)_2_(H_2_O)_4_·H_2_O, AP/Co(AFCA)_2_(H_2_O)_4_·H_2_O and AP/Fe(AFCA)_2_(H_2_O)_4_·H_2_O are increased by 2.16 mm s^−1^, 2.53 mm s^−1^, and 1.57 mm s^−1^ compared with the case of pure AP. This research shows considerable application prospects in improving the combustion and energy performance of solid propellants, it is also a reference for the design and preparation of other novel energetic catalysts.

## Abstract 2

Three energetic catalysts are designed and synthesized based on the coordination reaction of transition metal ions and an energetic ligand, which show considerable prospects in improving the combustion and energy performance of solid propellants.

## Graphical abstract

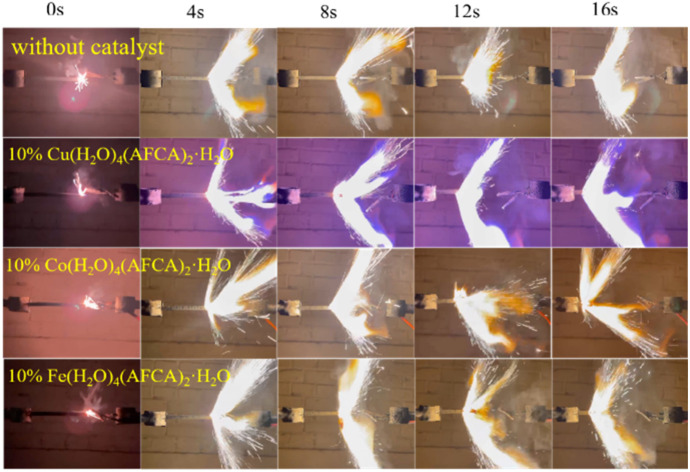

## Introduction

Composite solid propellants, composed of an oxidizer, additives, a binder, and fuels, are the most commonly used power source for rockets and missiles.^1^ Their energy and combustion performance are the most important factors influencing the properties of the solid rocket motor.^2,3^ An ideal composite solid propellant should possess a low-pressure exponent and an extremely stable burning rate.^4^ One of the most effective methods to achieve this goal is adding a combustion catalyst into the composite solid propellant to tune the ballistic properties of the rockets.^5^ Among them, developing new catalysts to promote the thermal decomposition of the oxidizer is considered as the simplest and most efficient strategy.^6^ Ammonium perchlorate (AP) is the most commonly used oxidant in composite solid propellants due to its advantages of high oxygen content, high density, low impact and friction sensitivity, and also its thermal decomposition characteristics without any solid residue.^7^ The mass content of AP in solid propellants is usually as high as 60% to 90%.^8^ Therefore, the development of catalysts to accelerate the thermal decomposition of AP is of great significance for improving the combustion and ballistic performance of composite solid propellant.

In order to reduce the thermal decomposition temperature of AP and increase its decomposition rate, researchers have carried out a lot of exploration in the past ten years.^9–11^ For example, numerous metal powders and alloys (Al, Ni, Cu, NiCu, *etc.*),^12,13^ transition metal oxides (Fe_2_O_3_, Co_3_O_4_, TiO_2_, CuO, Mn_2_O_3_, MnO_2_, *etc.*),^14–16^ composite metal oxide (NiFe_2_O_4_, ZnFe_2_O_4_, CoFe_2_O_4_, CuCo_2_O_4_),^17,18^ and carbon material^19,20^ have been evaluated for their catalytic performance in AP decomposition. These catalysts have a good effect on improving the thermal performance of AP. However, most of them are inert, which may lead to a decrease in the energy of solid propellant.^21^ Energetic catalysts can comprehensively improve the combustion performance and energy property of solid propellants. Preparation of catalysts with high energy density and strong catalytic activity has become the focus and frontier of research. In recent years, some energetic catalysts formed by metal ions and energetic compounds have been reported. For instance, energetic catalysts [Cu(atrz)_3_(NO_3_)_2_]_*n*_ (atrz = 4,4′-azo-1,2,4-triazole), [Cu(AT)_4_]Cl_2_ (AT = 5-amino-1*H*-tetrazole), [Cu(AzTO)(H_2_O)_3_]_*n*_, [Co(AzTO)(H_2_O)_4_·2H_2_O]_*n*_ (H_2_AzTO = 5,5′-azotetrazole-1,1′-diol), [Cu_2_(en)_2_(HBTI)_2_]_2_ (H_3_BTI = 4,5-bis(1*H*-tetrazol-5-yl)-1*H*-imidazole), [Co(en)(H_2_BTI)_2_]_2_·en *et al.*^3,22–24^ have been evaluated to reduce the thermal decomposition peak temperature and increase the heat release of AP. However, so far, the reports on energetic catalysts are very limited. More importantly, their effects on the combustion performance of propellants are rarely studied.

In this work, three energetic combustion catalysts were designed and prepared through the coordination reaction of transition metal ions (Cu^2+^, Co^2+^, Fe^2+^) and 3-aminofurazan-4-carboxylic acid (AFCA). All of them exhibit good effects on reducing the thermal decomposition temperature and activation energy of AP and improving the burning rate of solid propellant. AFCA is chosen as the energetic ligand because it has the following advantages: (1) furazan-ring has relatively high energy, its heat of formation (185 kJ mol^−1^) is approximately equal with that of tetrazole (200 kJ mol^−1^) and 1,2,4-triazole (182 kJ mol^−1^).^25^ (2) Furazan-ring is pentacyclic heterocyclic compound containing two potential coordination nitrogen atoms, one oxygen atom and two carbon atoms with relatively small volume, which may reduce steric hindrance and improve oxygen balance. (3) The ligand AFCA has abundant coordination modes, which can combine with metal ions to form a variety of spatial structures. (4) The energetic ligand AFCA has good thermal stability and low sensitivity. The Cu^2+^, Co^2+^, and Fe^2+^ are selected as the central ions because they usually have high catalytic activity for the thermal decomposition of AP. In addition, the effects of Cu^2+^, Co^2+^, and Fe^2+^ on the combustion performance of propellants can be studied through comparative experiments. In conclusion, the energetic catalysts designed in this study provide theoretical reference and experimental support for comprehensively improving the energy performance and combustion property of the solid propellant.

## Experimental section

Caution! The catalysts prepared in this study are energetic materials and tend to explode under certain conditions. Therefore, small-scale preparation is strongly encouraged. In addition, it is strongly recommended to operate in the hood behind a safety shield, and the eye protection and leather gloves must be worn.

### Materials

AFCA, Co(NO_3_)_2_·6H_2_O, Cu(NO_3_)_2_·3H_2_O, and FeCl_2_ were purchased from Energy Chemical Co., Ltd. with greater than 98% purity. NaOH, AP, hydroxyl-terminated polybutadiene (HTPB), dioctyl sebacate (DOS), Al powder, and CH_3_OH were purchased from Sigma-Aldrich Chemicals (St. Louis, MO), with greater than 98% purity. All chemicals were used as supplied, if not stated otherwise.

### Preparation procedures

#### Synthesis of energetic catalysts

**Scheme 1:**
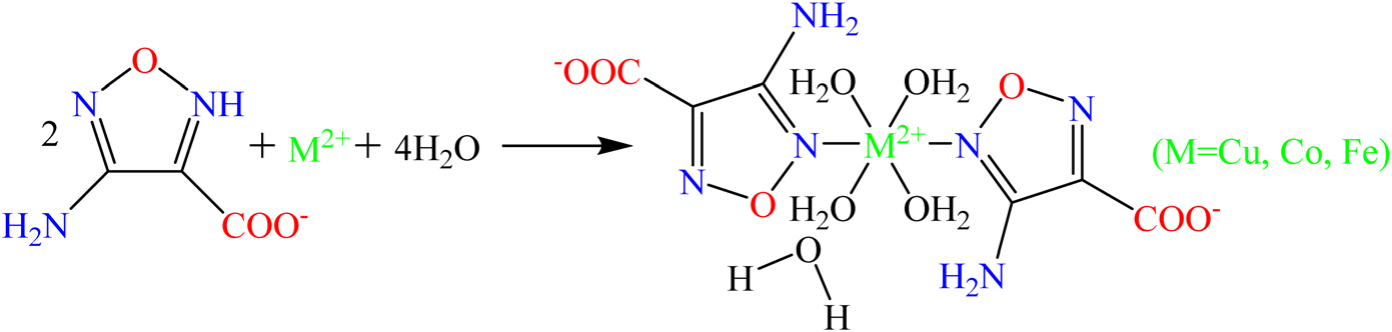
The synthesis path of [M(H_2_O)_4_(AFCA)_2_·H_2_O] (M = Cu, Co, Fe).

The synthesis path of energetic catalysts is shown in Scheme 1, and the specific preparation steps are listed below.

##### [Cu(H_2_O)_4_(AFCA)_2_·H_2_O]

Firstly, 0.258 g AFCA (2 mmol) and 0.08 g NaOH (2 mmol) were dissolved in 15 mL of distilled water at 70 °C. Then, the solution composed of 10 mL distilled water and 0.242 g Cu(NO_3_)_2_·3H_2_O (1 mmol) was slowly added to the above solution, and the mixture was stirred for 30 minutes at 70 °C. After that, the solution was cooled to 25 °C naturally and a deep blue precipitate was obtained. The precipitate was filtered off, washed with water and dried at 40 °C. The final product was in the form of blue fine powder (yield of 73%). Single crystals suitable for X-ray measurement were obtained by evaporation of the filtrate at room temperature for about one week.

##### [Co(H_2_O)_4_(AFCA)_2_·H_2_O]

Firstly, 0.258 g AFCA (2 mmol) and 0.08 g NaOH (2 mmol) were dissolved in 15 mL of distilled water at 70 °C. Then, the solution composed of 10 mL distilled water and 0.291 g Co(NO_3_)_2_·6H_2_O (1 mmol) was slowly added to the above solution, and the mixture was stirred for 30 minutes at 70 °C. After that, the solution was cooled to 25 °C naturally and a brown precipitate was obtained. The precipitate was filtered off, washed with water and dried at 40 °C. The final product was in the form of brown powder (yield of 78%).

The single crystals suitable for X-ray measurement were obtained by a diffusion method, its operation steps are described below. Firstly, 0.026 g AFCA (0.2 mmol) and 0.008 g NaOH (0.2 mmol) were added to 2 mL of distilled water to form a solution A. Then, 2.5 mL distilled water and 2.5 mL methanol were mixed to form solution B. After that, 0.029 g Co(NO_3_)_2_·6H_2_O (0.1 mmol) was dissolved in 2 mL methanol to form a solution C. Finally, solution A, solution B, and solution C were added into a clean glass tube in turn to form a diffusion system. The brown [Co(H_2_O)_4_(AFCA)_2_·H_2_O] crystals were obtained in the diffused part after seven days.

##### [Fe(H_2_O)_4_(AFCA)_2_·H_2_O]

Firstly, 0.258 g AFCA (2 mmol) and 0.08 g NaOH (2 mmol) were dissolved in 15 mL of distilled water at 70 °C. Then, the solution composed of 10 mL distilled water and 0.127 g FeCl_2_ (1 mmol) was slowly added to the above solution, and the mixture was stirred for 30 minutes at 70 °C. After that, the solution was cooled to 25 °C naturally and a yellow precipitate was obtained. The precipitate was filtered off, washed with water and dried at 40 °C. The final product was in the form of yellow powder (yield of 71%).

The single crystals suitable for X-ray measurement were obtained by a diffusion method, its operation steps are described below. Firstly, 0.026 g AFCA (0.2 mmol) and 0.008 g NaOH (0.2 mmol) were added to 2 mL of distilled water to form a solution A. Then, 2.5 mL distilled water and 2.5 mL methanol were mixed to form solution B. After that, 0.013 g FeCl_2_ (0.1 mmol) was dissolved in 2 mL methanol to form a solution C. Finally, solution A, solution B, and solution C were added into a clean glass tube in turn to form a diffusion system. The yellow [Fe(H_2_O)_4_(AFCA)_2_·H_2_O] crystals were obtained in the diffused part after seven days.

### Preparation of solid propellent

Composite solid propellant containing AP and energetic combustion catalyst was prepared by a pouring and solidification method. Firstly, 13.03 g HTPB, 0.97 g TDI, 3.00 g DOS, 20 g Al powder, 59.22 g AP, and 3.78 g energetic catalyst were mixed in a stirred reactor coupled to a thermostatic water bath circulator set at 40 °C. Then, the mixture was poured into the mold and left under vacuum for 20 min. Finally, the mold was then left in an oven at 70 °C to cure for one week, and the solid propellant grain with the size of 6 mm × 6 mm × 10 mm can be obtained.

### General methods

The prepared crystals were mounted on a Bruker D8 VENTURE diffractometer using Mo-Kα radiation (*λ* = 0.71073 Å) with a graphite monochromator at 170 K. Integration and scaling of intensity data was performed using the SAINT program. Data was corrected for the effects of absorption using SADABS. The structure was solved by direct method and refined with full-matrix least-squares technique using SHELX-2014 software. Non-hydrogen atoms were refined with anisotropic displacement parameters, and hydrogen atoms were placed in calculated positions and refined with a riding model. The differential thermal analysis (DTA, HCT-4) tests were performed at a heating rate of 10 °C min^−1^ in closed ceramic containers with a high-purity argon flow of 30 mL min^−1^. Powder X-ray diffraction (PXRD) tests were performed on a Bruker D8 Advance X-ray diffractometer using Cu Kα (*λ* = 1.5406 Å) radiation. Fourier transform infrared spectroscopy (FTIR) spectra were measured by using KBr pellets for samples from 4000 cm^−1^ to 400 cm^−1^ on a Thermo Nicolet iS10 spectrometer. The morphologies of the samples were characterized with FEI field-emission scanning electron microscope (SEM, Quanta 250F) equipped with an energy dispersive X-ray spectrometer (EDS). The combustion performance of solid propellent was recorded by a camera (OSG030-790UMTZ) running at 1000 frames per second.

## Results and discussion

### Design of energetic catalysts

**Fig. 1:**
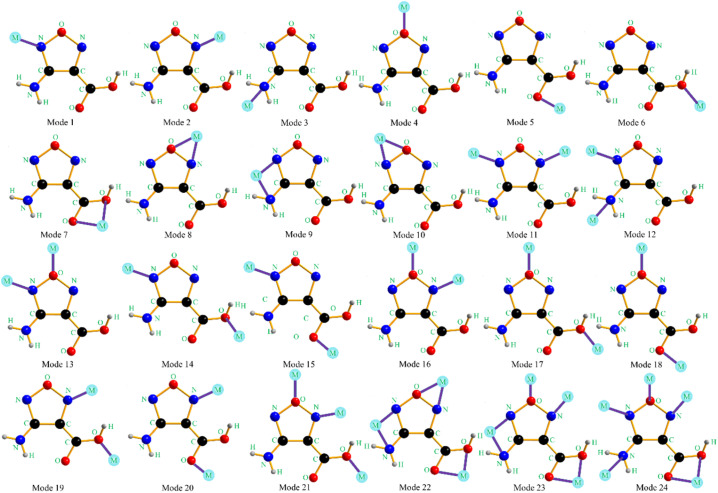
The potential coordination modes of AFCA and transition metal ions.

The design of energetic combustion catalysts mainly considers catalytic activity and energy performance. It is reported that transition metal ions have good catalytic effect on the thermal decomposition of AP.^16,18^ In addition, some nitrogen heterocyclic compounds have excellent energy performance. Therefore, the assembly of nitrogen heterocyclic energetic compounds and transition metal ions through coordination bonds or ionic bonds provides technical ways for the design of energetic combustion catalysts. In this study, Cu^2+^, Co^2+^ and Fe^2+^ are selected as the central ions because they are green and have better catalytic effects than other transition metal ions such as Ag^+^, Cd^2+^, Zn^2+^, and Pb^2+^. In addition, AFCA is selected as the energetic ligand. On the one hand, furazan ring has good energy performance, and its heat of formation is up to 185 kJ mol^−1^. On the other hand, as shown in Fig. 1, AFCA has rich potential coordination sites and can combine with metal ions to form a variety of spatial structures.

### Crystal structure of energetic catalysts

**Fig. 2:**
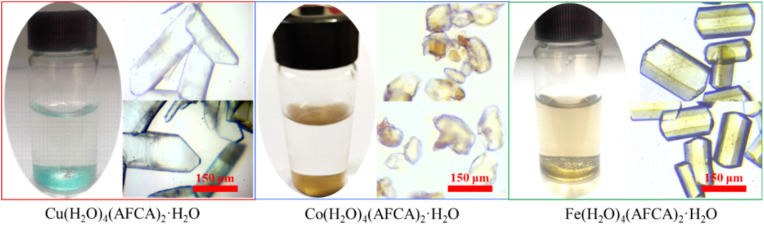
The microscope images of as-prepared energetic catalysts.

In order to characterize the crystal structure of as-prepared energetic catalysts, the solvent evaporation method and liquid diffusion method are used to cultivate single crystals. As shown the morphology in Fig. 2, the shape of Cu(AFCA)_2_(H_2_O)_4_·H_2_O is light blue strip with an average length of 364 μm. In addition, Co(AFCA)_2_(H_2_O)_4_·H_2_O exhibits an irregular block shape, and its maximum particle size can reach 155 μm. The morphology of Fe(AFCA)_2_(H_2_O)_4_·H_2_O is uniform short rod with the particle size of 215 μm.

**Fig. 3:**
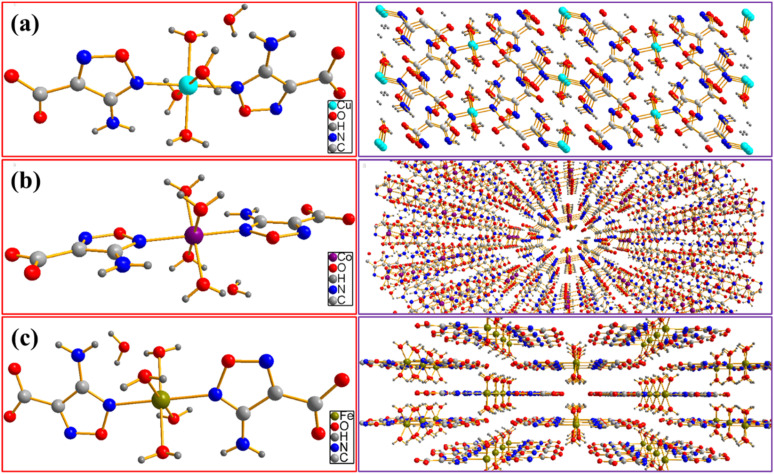
Molecular structure and packing plot of (a) Cu(AFCA)_2_(H_2_O)_4_·H_2_O, (b) Co(AFCA)_2_(H_2_O)_4_·H_2_O, and (c) Fe(AFCA)_2_(H_2_O)_4_·H_2_O.

The crystal structures of as-prepared energetic catalysts are determined by X-ray crystallography. As shown in Table 1, Cu(AFCA)_2_(H_2_O)_4_·H_2_O crystallizes in the monoclinic crystal system with space group *P*_21/*n*_, *a* = 6.4481(10) Å, *b* = 17.558(3) Å, *c* = 6.5592(10) Å, *D*_c_ = 1.918 g cm^−3^. The corresponding crystal structure is shown in Fig. 3a, it can be clearly seen that the asymmetric unit of Cu(AFCA)_2_ (H_2_O)_4_·H_2_O shows a octahedral arrangement, which consists of one crystallographically independent Cu^II^ center, two AFCA ligands, four coordinated water molecules and one uncoordinated water molecule. In addition, the distance of Cu–N bond (2.022 Å) is between that of Cu–O1 bond (1.976 Å) and Cu–O2 bond (2.313 Å) in Cu(AFCA)_2_(H_2_O)_4_·H_2_O. The bond angles of two N (or O) atoms of two contraposition AFCA (or H_2_O) ligands and Cu^II^ cation are all 180° (ESI Tables S1 and S2†).

**Table 1:** Crystallographic data for the prepared energetic catalysts

Compound	Cu(AFCA)_2_(H_2_O)_4_·H_2_O	Co(AFCA)_2_(H_2_O)_4_·H_2_O	Fe(AFCA)_2_(H_2_O)_4_·H_2_O
CCDC	1534613	1534658	1534612
Chemical formula	C_6_H_16_CuN_6_O_12_	C_6_H_16_CoN_6_O_12_	C_6_H_16_FeN_6_O_12_
Formula weight/g mol^−1^	427.79	423.18	420.10
Crystal system	Monoclinic	Orthorhombic	Orthorhombic
Space group	*P21*/*n*	*Pnnm*	*Pnnm*
Temperature (K)	170.0	293(2)	170.0
*a*/Å	6.4481(10)	6.4342(7)	17.768(4)
*b*/Å	17.558(3)	17.7202(19)	6.4114(12)
*c*/Å	6.5592(10)	6.5370(7)	6.5376(13)
*α*/°	90	90	90
*β*/°	94.045(4)	90	90
*γ*/°	90	90	90
Volume/Å^3^	740.74(19)	745.32(14)	744.8(3)
*Z*	2	2	2
Calculated density (g cm^−3^)	1.918	1.886	1.873
*μ* (mm^−1^)	1.557	1.233	1.095
*F*(000)	438	434	432.0
Reflections collected/unique	6351/1679	9088/723	4876/927
Reflections collected	969	892	4876
Goodness-of-fit on *F*^2^	1.157	1.054	1.033
Data/restraints/parameters	1679/0/120	723/0/74	927/12/78
Goodness-of-fit on *F*^2^	1.194	1.028	1.033
Final *R* indices [*I* > 2sigma(*I*)]	*R*_1_ = 0.0752, w*R*_2_ = 0.1997	*R*_1_ = 0.0376, w*R*_2_ = 0.1019	*R*_1_ = 0.0401, w*R*_2_ = 0.0933
*R* Indices (all data)	*R*_1_ = 0.0822, w*R*_2_ = 0.2061	*R*_1_ = 0.0386, w*R*_2_ = 0.1040	*R*_1_ = 0.0625, w*R*_2_ = 0.1026
Largest peak and hole (e Å^−3^)	2.34 and −0.45	1.581 and −0.529	0.41 and −0.55

In addition, Co(AFCA)_2_(H_2_O)_4_·H_2_O and Fe(AFCA)_2_(H_2_O)_4_·H_2_O crystallize in the orthorhombic crystal system, pertain to space group *Pnnm*. As for the crystal parameters of Co(AFCA)_2_(H_2_O)_4_·H_2_O, *a* = 6.4342(7) Å, *b* = 17.7202(19) Å, *c* = 6.5370(7) Å, *D*_c_ = 1.886 g cm^−3^, which is approximately equal with that of Fe(AFCA)_2_(H_2_O)_4_·H_2_O (*a* = 6.4315(7) Å, *b* = 17.7587(18) Å, *c* = 6.5831(7) Å, *D*_c_ = 1.856 g cm^−3^). As shown in Fig. 3b and c, the Co(AFCA)_2_(H_2_O)_4_·H_2_O and Fe(AFCA)_2_(H_2_O)_4_·H_2_O exhibit similar structure with that of Cu(AFCA)_2_(H_2_O)_4_·H_2_O. Each asymmetric unit shows an appreciably distorted-octahedral configuration, which is composed of one metal ion center, two AFCA ligands, four coordinated water molecules and one uncoordinated water molecule. In addition, each furazan-ring shows monodentate coordination mode and the carboxyl group does not participate in the coordination. As shown in ESI Tables S3 and S4,† the distance of Co–N bond and Co–O1 bond in Co(AFCA)_2_(H_2_O)_4_·H_2_O are 2.137 Å and 2.0769 Å, respectively. The bond angles of two N (or O) atoms of two contraposition AFCA (or H_2_O) ligands and Co^II^ cation are all 180°. Moreover, as shown in ESI Tables S5 and S6,† the distance of Fe–N bond and Co–O1 bond in Fe(AFCA)_2_(H_2_O)_4_·H_2_O are 2.155 Å and 2.1139 Å, respectively. The bond angles of two N (or O) atoms of two contraposition AFCA (or H_2_O) ligands and Fe^II^ cation are all 180°.

### FTIR and XRD characterization

**Fig. 4:**
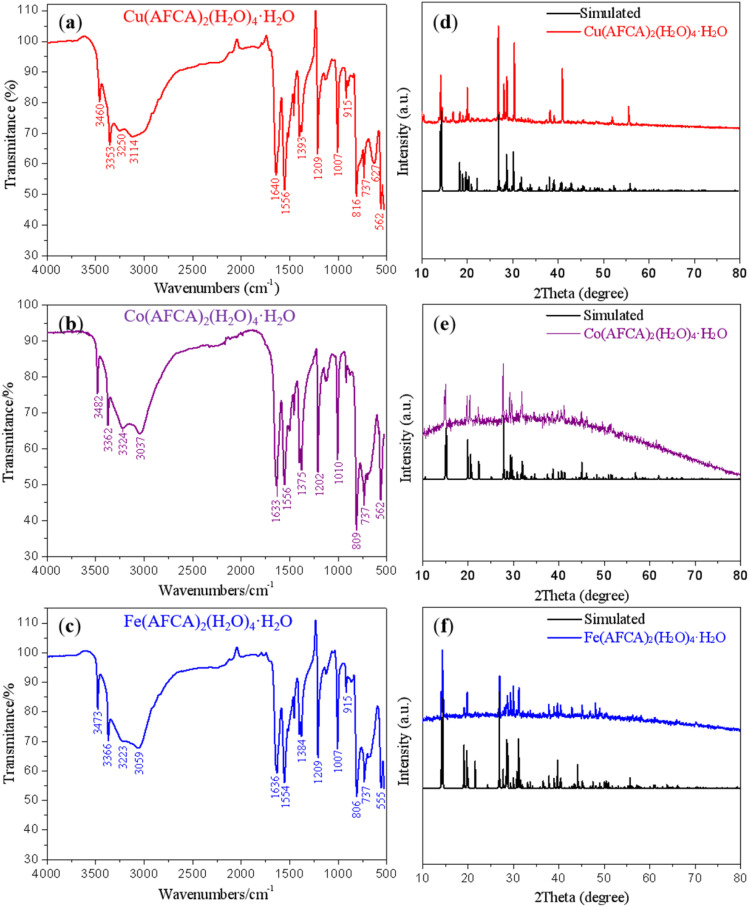
FTIR and PXRD spectra patterns of prepared samples. (a–c) represent the FTIR spectra of Cu(AFCA)_2_(H_2_O)_4_·H_2_O, Co(AFCA)_2_(H_2_O)_4_·H_2_O, and Fe(AFCA)_2_(H_2_O)_4_·H_2_O respectively. (d–f) represent the PXRD spectra of Cu(AFCA)_2_(H_2_O)_4_·H_2_O, Co(AFCA)_2_(H_2_O)_4_·H_2_O, and Fe(AFCA)_2_(H_2_O)_4_·H_2_O respectively.

FTIR and PXRD analyses were also performed to further confirm the structure and phase purity of prepared samples. As shown in Fig. 4a–c, the stretching vibration peaks of Cu(AFCA)_2_(H_2_O)_4_·H_2_O basically match that of Co(AFCA)_2_(H_2_O)_4_·H_2_O and Fe(AFCA)_2_(H_2_O)_4_·H_2_O, except for some minor differences in peak positions. This is because the coordination bonds formed by different metal ions affect the infrared absorption of functional groups. In the FTIR spectrum of Cu(AFCA)_2_(H_2_O)_4_·H_2_O, the absorption bands at 3114 cm^−1^ and 3250 cm^−1^ belong to the characteristic vibrations of crystal water, whereas infrared absorption peaks at 3460 cm^−1^, 3353 cm^−1^ are ascribed to the characteristic vibrations of –NH_2_. Moreover, three strong vibrations peaks located at 1640 cm^−1^, 1556 cm^−1^, and 1393 cm^−1^ belong to the characteristic absorption peaks of furazan ring, while infrared absorption peaks at 1007 cm^−1^, 915 cm^−1^, and 816 cm^−1^ are ascribed to the characteristic vibrations of C=N and N–O. In addition, the FTIR spectra patterns of Co(AFCA)_2_(H_2_O)_4_·H_2_O and Fe(AFCA)_2_(H_2_O)_4_·H_2_O show the similar results with that of Cu(AFCA)_2_(H_2_O)_4_·H_2_O. The phase purity and crystallinity of as-prepared samples are confirmed by the similarity between the experimental and simulated PXRD patterns. As shown in Fig. 4d–f, the PXRD patterns of the three compounds are consistent with that of the simulated patterns. These results show that the designed energetic combustion catalysts are successfully prepared.

### Thermal analysis and catalytic performance characterization

**Fig. 5:**
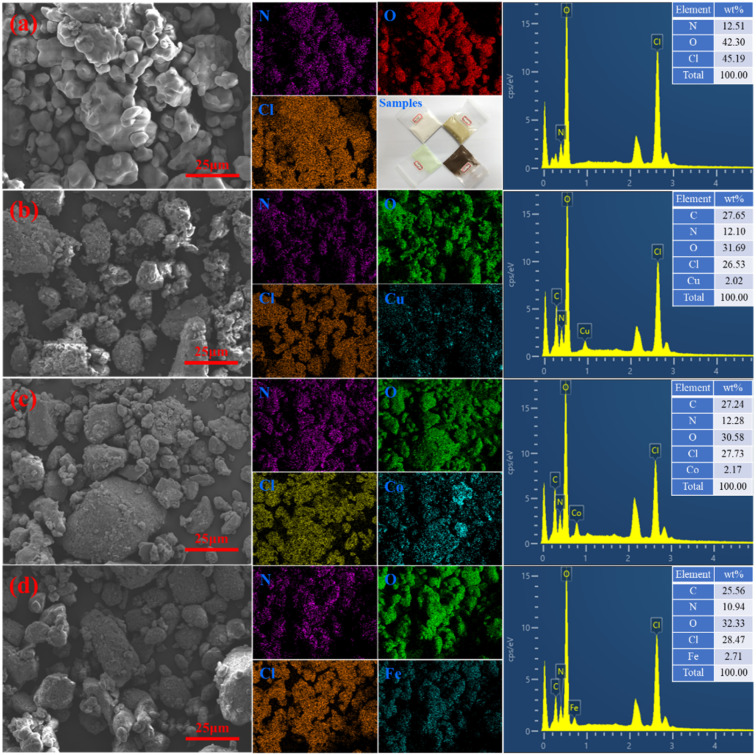
The morphologies of (a) AP raw material, (b) AP/Cu(AFCA)_2_(H_2_O)_4_·H_2_O (94 : 6), (c) AP/Co(AFCA)_2_(H_2_O)_4_·H_2_O (94 : 6), (d) AP/Fe(AFCA)_2_(H_2_O)_4_·H_2_O (94 : 6).

In order to characterize the thermal performance and catalytic activity of the prepared samples, AP and M(AFCA)_2_(H_2_O)_4_·H_2_O (M = Cu, Co, Fe) are mixed evenly by physical grinding method in the proportion of 98 : 2, 94 : 6, and 90 : 10, respectively. Fig. 5 shows the morphology of the mixture when the catalyst content is 6%. It can be clearly seen that the shape of AP raw material is ellipsoidal, and its chemical composition only contains N, H, Cl and O. The morphology of AP/M(AFCA)_2_(H_2_O)_4_·H_2_O (M = Cu, Co, Fe) mixture is basically the same as that of AP raw material, except that its surface is uniformly coated by energetic catalyst. The EDS spectrum in Fig. 5b–d further confirms this conclusion. It is not difficult to see that the transition metal elements (Cu, Co, Fe) are uniformly distributed on the surface of the prepared samples, and their contents account for 2.02%, 2.17% and 2.71% respectively. These results indicate that the prepared catalysts are evenly dispersed on the surface of AP particles.

**Fig. 6:**
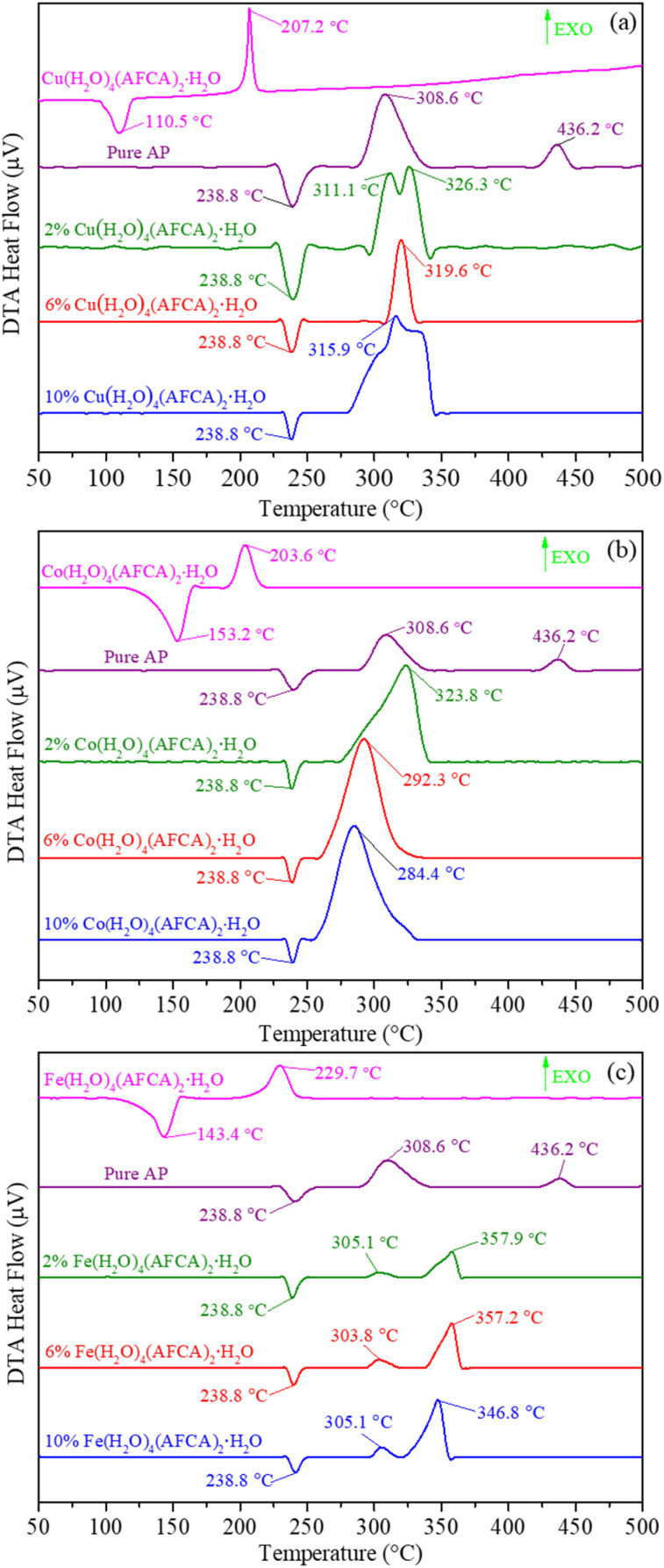
The DTA curves of (a) AP, Cu(AFCA)_2_(H_2_O)_4_·H_2_O and AP/Cu(AFCA)_2_(H_2_O)_4_·H_2_O; (b) AP, Co(AFCA)_2_(H_2_O)_4_·H_2_O and AP/Co(AFCA)_2_(H_2_O)_4_·H_2_O; (c) AP, Fe(AFCA)_2_(H_2_O)_4_·H_2_O and AP/Fe(AFCA)_2_(H_2_O)_4_·H_2_O.

The DTA curves of the pure AP, as-prepared energetic catalysts, and AP/M(AFCA)_2_(H_2_O)_4_·H_2_O (M = Cu, Co, Fe) mixtures are shown in Fig. 6. It is not hard to find an obvious endothermic peak and exothermic peak in the DTA curve of each catalyst. Among them, the endothermic peaks located at 110.5 °C, 153.2 °C and 143.4 °C are attributed to the loss of crystal water from M(AFCA)_2_(H_2_O)_4_·H_2_O (M = Cu, Co, Fe), while the exothermic peaks located at 207.2 °C, 203.6 °C and 229.7 °C correspond to the decomposition exothermic of energetic ligand. In addition, as shown in Fig. 6a–c, the endothermic peaks located at 238.8 °C are attributed to the phase transition of AP from orthorhombic to cubic form due to the rotation of perchlorate ions.^26^ The thermal decomposition process of pure AP goes through two stages. The first stage is usually called low temperature decomposition (LTD) stage, the exothermic peak of which appears at more than 300 °C and the decomposition products are O_2_, H_2_O, N_2_O, Cl_2_, NO, *etc.* The second stage corresponds to the high temperature decomposition (HTD) stage, in which the exothermic peak appears above 400 °C and the intermediate product decomposes completely.^6,27^ For AP/Cu(AFCA)_2_(H_2_O)_4_·H_2_O, when the content of catalyst is 2%, the thermal decomposition process also goes through two stages. Its peak temperatures are 311.1 °C and 326.3 °C respectively, which are significantly lower than that of the pure AP. However, when the content of Cu(AFCA)_2_(H_2_O)_4_·H_2_O is increased to 6% and 10%, only one exothermic peak appears in the DTA curve of each sample. Their thermal decomposition peak temperatures are 319.6 °C and 315.9 °C, respectively, which are 116.6 °C and 120.3 °C lower than that of the pure AP. The thermal decomposition process of AP/Co(AFCA)_2_(H_2_O)_4_·H_2_O also tends to go through one stage. As shown in Fig. 6b, when the catalyst content is 2%, 6%, and 10%, only one exothermic peak appears in the DTA curve of each sample. Their thermal decomposition peak temperatures are 323.8 °C, 292.3 °C, and 284.4 °C, respectively, which are 112.4 °C, 143.9 °C, and 151.8 °C lower than that of the pure AP. However, different from the case of AP/M(AFCA)_2_(H_2_O)_4_·H_2_O (M = Cu, Co), the thermal decomposition process of AP/Fe(AFCA)_2_(H_2_O)_4_·H_2_O tends to go through two stages. As shown in Fig. 6c, when the content of Fe(AFCA)_2_(H_2_O)_4_·H_2_O is 2%, 6%, and 10%, the LTD peak temperature of AP/Fe(AFCA)_2_(H_2_O)_4_·H_2_O is around 305 °C, which is slightly lower than that of pure AP (308.6 °C). While the corresponding HTD peak temperatures of AP/Fe(AFCA)_2_(H_2_O)_4_·H_2_O are 357.9 °C, 357.2 °C and 346.8 °C, respectively, which are reduced by 78.3 °C, 79.0 °C and 89.5 °C compared to pure AP. These results show that the designed energetic catalysts have excellent catalytic effects on the thermal decomposition of AP. Compared with the catalysts reported in literature,^23,24,28^ M(AFCA)_2_(H_2_O)_4_·H_2_O (M = Cu, Co) exhibits more excellent catalytic activity. As shown in Table 2, when the content of catalyst is 10%, the thermal decomposition peak temperature of AP with the addition of Cu(AFCA)_2_(H_2_O)_4_·H_2_O (315.9 °C) and Co(AFCA)_2_(H_2_O)_4_·H_2_O (284.4 °C) is significantly lower than that with the addition of [Co(en)(H_2_BTI)_2_]_2_·en (333.7 °C), [Cu_2_(en)_2_(HBTI)_2_]_2_ (336.1 °C), Co-AzTO (350.8 °C), Ni-AzTO (383.8 °C), and [Pb(BTF)(H_2_O)_2_]_*n*_ (344.0 °C). However, the catalytic effect of Fe(AFCA)_2_(H_2_O)_4_·H_2_O does not show significant advantages. Its catalytic activity is comparable to that of Co-AzTO and [Pb(BTF)(H_2_O)_2_]_*n*_, but lower than that of [Co(en)(H_2_BTI)_2_]_2_·en, [Cu_2_(en)_2_(HBTI)_2_]_2_, and Cu-AzTO. Moreover, with the same additive amount of catalyst, Co(AFCA)_2_(H_2_O)_4_·H_2_O has the greatest effect on the thermal decomposition temperature of AP, followed by Cu(AFCA)_2_(H_2_O)_4_·H_2_O and Fe(AFCA)_2_(H_2_O)_4_·H_2_O. For example, when the content of catalyst is 10%, the addition of Co(AFCA)_2_(H_2_O)_4_·H_2_O, Cu(AFCA)_2_(H_2_O)_4_·H_2_O, and Fe(AFCA)_2_(H_2_O)_4_·H_2_O leads to a decrease in the HTD peak temperature of AP by 120.3 °C, 151.8 °C, and 89.5 °C, respectively. These results indicate that Co(AFCA)_2_(H_2_O)_4_·H_2_O has the highest catalytic activity, followed by Cu(AFCA)_2_(H_2_O)_4_·H_2_O and Fe(AFCA)_2_(H_2_O)_4_·H_2_O.

**Table 2:** Catalytic performance of various additives in the thermal decomposition of AP^a^

Catalyst	Percentage (%)	Peak temperature (°C)
Cu(AFCA)_2_(H_2_O)_4_·H_2_O	10	315.9
Co(AFCA)_2_(H_2_O)_4_·H_2_O	10	284.4
Fe(AFCA)_2_(H_2_O)_4_·H_2_O	10	346.8
[Co(en)(H_2_BTI)_2_]_2_·en	10	333.7 (ref. 23)
[Cu_2_(en)_2_(HBTI)_2_]_2_	10	336.1 (ref. 23)
Co-AzTO	10	350.8 (ref. 24)
Ni-AzTO	10	383.8 (ref. 24)
Cu-AzTO	10	298.9 (ref. 24)
[Pb(BTF)(H_2_O)_2_]_*n*_	10	344.0 (ref. 28)

a H_3_BTI = 4,5-bis(1*H*-tetrazol-5-yl)-1*H*-imida-zole, en = ethylenedi-amine, H_2_AzT = 5,5′-azotetrazole-1,1′-diol, BTF = 4,4′-oxybis [3,3′-(1-hydroxy-tetrazolyl)]furazan.

**Scheme 2:**
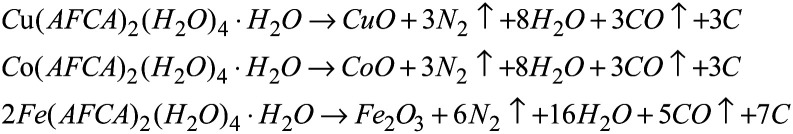


The above results can be explained by the catalytic theory of transition metal ions for AP. As shown in Fig. 6, when the prepared samples are heated to 245 °C, the energetic catalysts M(AFCA)_2_(H_2_O)_4_·H_2_O (M = Cu, Co, Fe) are substantially completely decomposed, while AP has not start to decompose at that temperature. According to the largest exothermic principle,^29^ the chemical reaction equations for the thermal decomposition of M(AFCA)_2_(H_2_O)_4_·H_2_O (M = Cu, Co, Fe) are listed in Scheme 2.

**Fig. 7:**
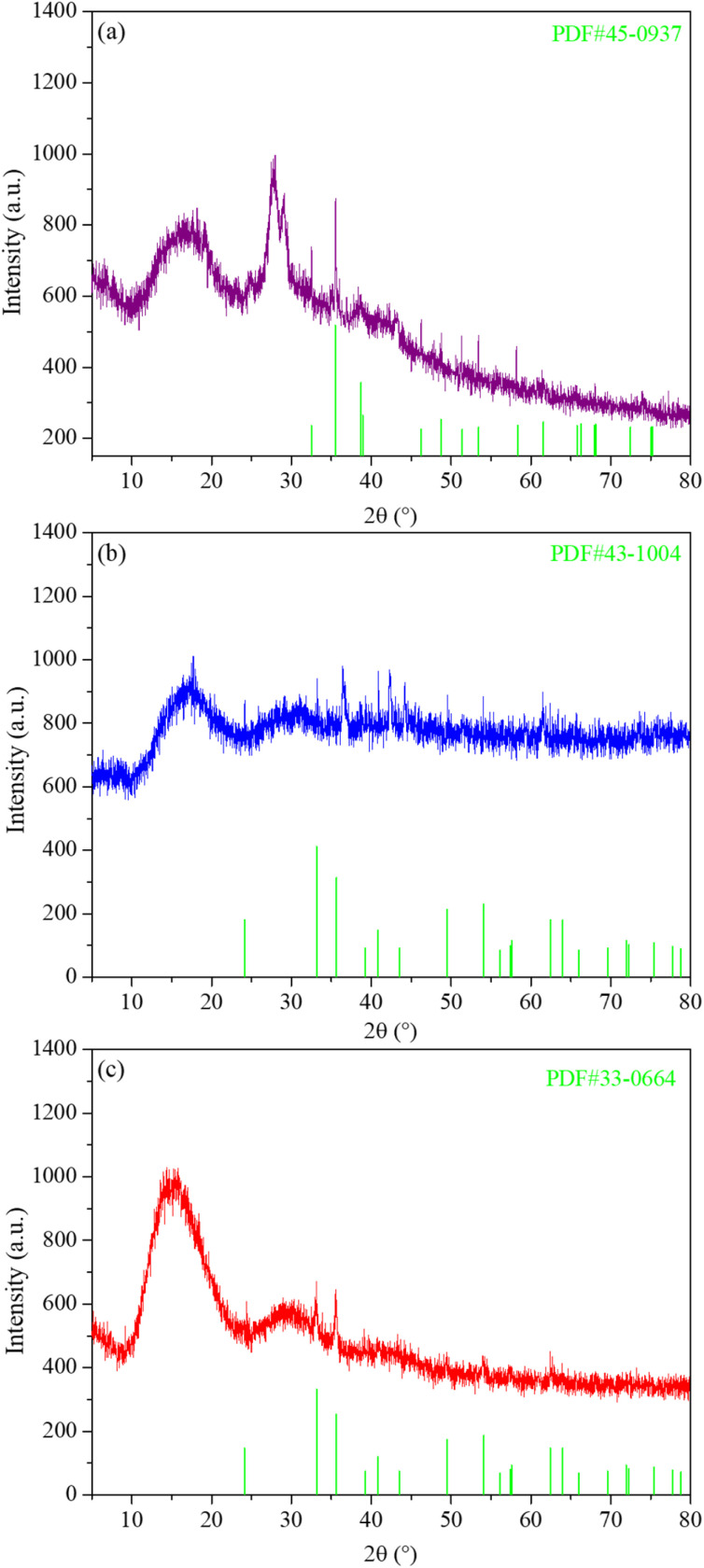
PXRD spectra of thermal decomposition products of (a) Cu(AFCA)_2_(H_2_O)_4_·H_2_O, (b) Co(AFCA)_2_(H_2_O)_4_·H_2_O, and (c) Fe(AFCA)_2_(H_2_O)_4_·H_2_O.

**Scheme 3:**
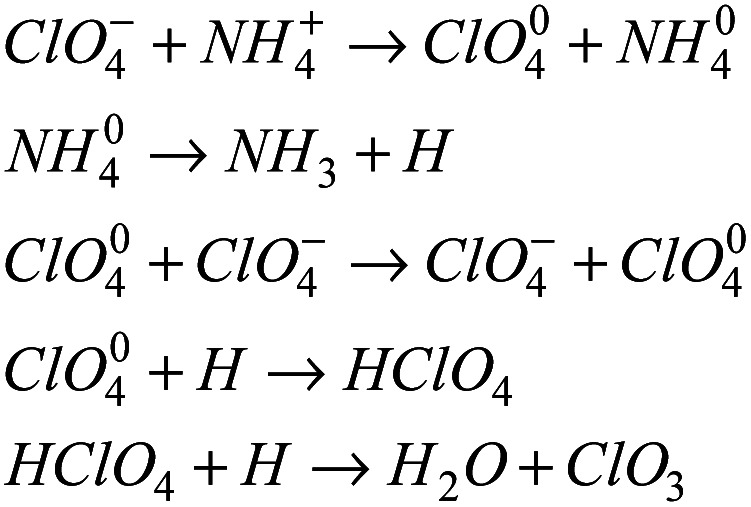


Since the energetic ligand contains oxygen atoms, the main solid products of M(AFCA)_2_(H_2_O)_4_·H_2_O (M = Cu, Co, Fe) after decomposition are composed of carbon and transition metal oxides (such as CuO, CoO, Fe_2_O_3_). These can be verified by PXRD characterization. As shown in Fig. 7, the diffraction pattern of the solid product of Cu(AFCA)_2_(H_2_O)_4_·H_2_O thermally decomposed in argon atmosphere shows diffraction peaks located at 32.5°, 35.5°, 38.7°, 46.2°, 48.8°, 51.2°, 53.5°, and 58.2° match the diffraction peak positions of CuO (JCPDS card no. 45-0937). In addition, the PXRD spectra of solid products of Co(AFCA)_2_(H_2_O)_4_·H_2_O and Fe(AFCA)_2_(H_2_O)_4_·H_2_O are consistent with the diffraction peak positions of CoO (JCPDS Card no. 43-1004) and Fe_2_O_3_ (JCPDS card no. 33-0664), respectively (Fig. 7b and c). These results prove the formation of CuO, CoO, and Fe_2_O_3_ particles. However, when the temperature of the sample is raised to around 300 °C, the AP particles begin to decompose. As reported in the literature,^30–32^ the first decomposition step is solid–gas multiphase reaction, including decomposition and sublimation (Scheme 3). Meanwhile, a series of reactions happen to produce large amount of N_2_O, O_2_, Cl_2_, H_2_O, HCl and a small amount of NO.

**Scheme 4:**
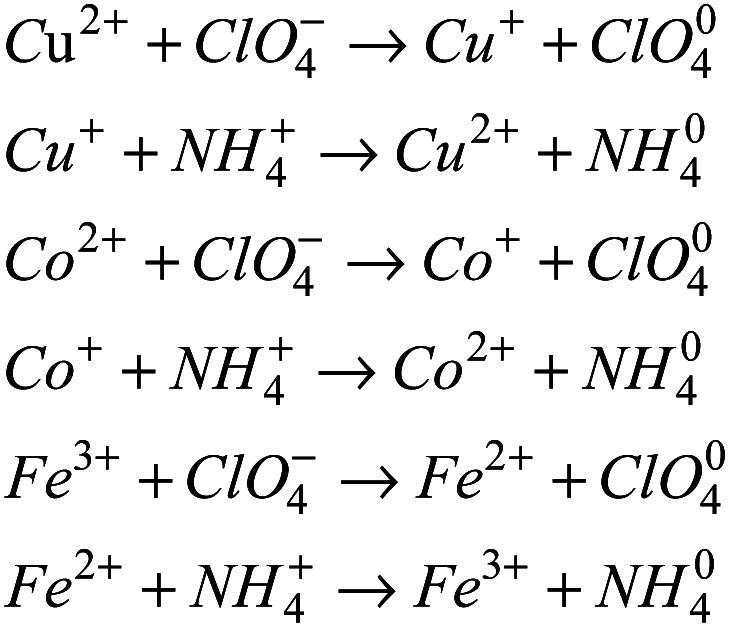


In addition, with the further increase of temperature, the ClO_3_ radical traps an electron to generate a chlorate ion, which can further decompose or interact with ammonium ions. According to the electron transfer theory,^32,33^ the generated CuO, CoO, and Fe_2_O_3_ can provide a bridge for the transfer of electrons from the ClO_4_^−^ to NH_4_^+^ during the decomposition process of AP, thereby reducing the thermal decomposition temperature of AP (Scheme 4).

**Fig. 8:**
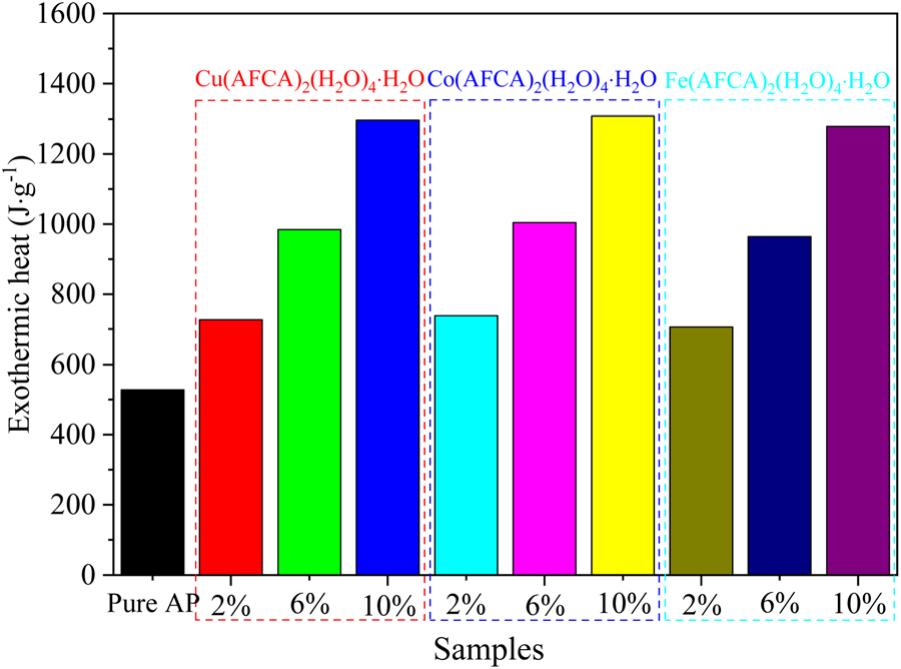
The heat release of pure AP and as-prepared AP/M(H_2_O)_4_(AFCA)_2_·H_2_O (M = Cu, Co, Fe) samples.

In addition to reducing the thermal decomposition temperature of AP, the prepared energetic catalysts are also conducive to improve the thermal decomposition heat release. As shown in Fig. 8, when the content of energetic catalyst is 2%, the heat release of AP/Cu(AFCA)_2_(H_2_O)_4_·H_2_O, AP/Co(AFCA)_2_(H_2_O)_4_·H_2_O and AP/Fe(AFCA)_2_(H_2_O)_4_·H_2_O are 726.4 J g^−1^, 738.1 J g^−1^, 706.6 J g^−1^ respectively, which are significantly higher than that of pure AP (527.7 J g^−1^). Moreover, with the increase of catalyst content, the heat release of the AP/M(H_2_O)_4_(AFCA)_2_·H_2_O (M = Cu, Co, Fe) samples tend to increase. When the content of catalyst is increased to 6% and 10% respectively, the heat release of AP/Cu(AFCA)_2_(H_2_O)_4_·H_2_O, AP/Co(AFCA)_2_(H_2_O)_4_·H_2_O and AP/Fe(AFCA)_2_(H_2_O)_4_·H_2_O are increased to 984.3 J g^−1^, 1004.8 J g^−1^, 964.2 g^−1^ and 1296.5 J g^−1^, 1308.2 J g^−1^, 1278.6 J g^−1^, respectively. These results indicate that the energetic catalysts designed and prepared in this study not only have excellent catalytic activity for the thermal decomposition of AP, but also can significantly increase its heat release.

### Combustion performance test

**Fig. 9:**
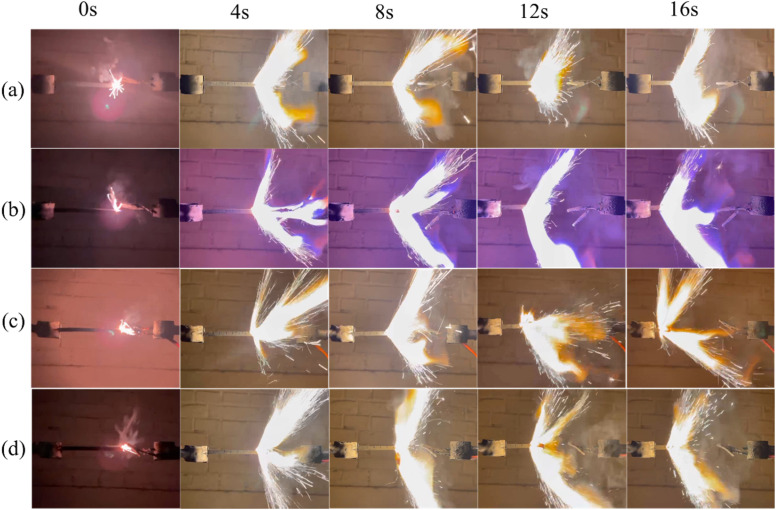
The combustion process of solid propellent with different type and content of catalyst. (a) Pure AP, (b) AP/Cu(H_2_O)_4_(AFCA)_2_·H_2_O, (c) AP/Co(H_2_O)_4_(AFCA)_2_·H_2_O, (d) AP/Fe(H_2_O)_4_(AFCA)_2_·H_2_O.

**Fig. 10:**
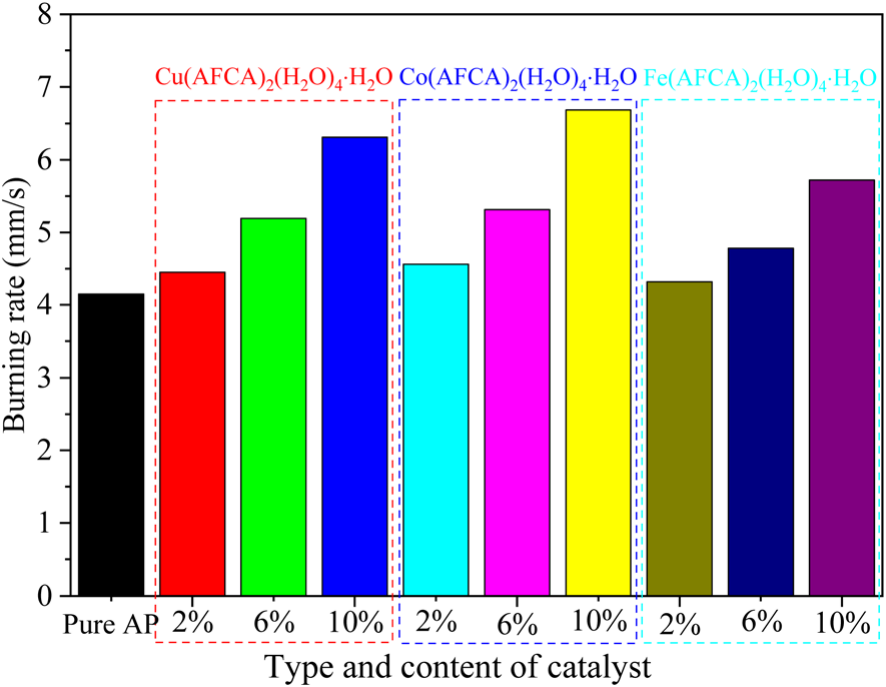
The burning rate of solid propellent with different type and content of catalyst.

The effect of the prepared catalysts on the combustion performance of solid propellant is also investigated. Herein, the pouring process is adopted to prepare the solid propellents composed of AP/M(H_2_O)_4_(AFCA)_2_·H_2_O (M = Cu, Co, Fe), Al, HTPB, DOS, and TDI (63.00 : 20.00 : 13.03 : 3.00 : 0.97), and the size of the propellant strip is 6 mm × 6 mm × 100 mm. The propellant strip is ignited by the Ni–Cr wire, and its working voltage and current are 2.00 V and 1.32 mA, respectively. The combustion process of the solid propellent is recorded by the camera, and the corresponding linear burning rate is obtained by converting the length of the grain burned per second. As shown the results in Fig. 9, all samples can self-propagating combustion and emit bright white light. Among them, the propellant containing Cu(H_2_O)_4_(AFCA)_2_·H_2_O catalyst in the component produces a blue flame when burning. While the propellants containing Co(H_2_O)_4_(AFCA)_2_·H_2_O and Fe(H_2_O)_4_(AFCA)_2_·H_2_O in the component produce a yellow flame when burning, this is due to the different flame reaction of Cu^2+^, Co^2+^ and Fe^2+^ ions. In addition, it can also be seen from Fig. 9 that the length of solid propellent grain burned in the same time is also different. As shown the test results in Fig. 10, when the content of energetic catalyst is 2%, the burning rate of solid propellent composed of AP/Cu(AFCA)_2_(H_2_O)_4_·H_2_O, AP/Co(AFCA)_2_(H_2_O)_4_·H_2_O and AP/Fe(AFCA)_2_(H_2_O)_4_·H_2_O are 4.45 mm s^−1^, 4.56 mm s^−1^, 4.32 mm s^−1^ respectively, which are higher than that of the pure AP case (4.15 mm s^−1^). Moreover, with the increase of catalyst content, the burning rate of the solid propellent tends to increase. When the content of catalyst is increased to 6% and 10% respectively, the burning rate of solid propellent composed of AP/Cu(AFCA)_2_(H_2_O)_4_·H_2_O, AP/Co(AFCA)_2_(H_2_O)_4_·H_2_O and AP/Fe(AFCA)_2_(H_2_O)_4_·H_2_O are increased to 5.19 mm s^−1^, 5.31 mm s^−1^, 4.78 mm s^−1^ and 6.31 mm s^−1^, 6.68 mm s^−1^, 5.72 mm s^−1^, respectively. Furthermore, it can be seen from Fig. 10 that Co(AFCA)_2_(H_2_O)_4_·H_2_O has the best effect on improving the burning rate of solid propellant, followed by Cu(AFCA)_2_(H_2_O)_4_·H_2_O and Fe(AFCA)_2_(H_2_O)_4_·H_2_O, which are consistent with the results of thermal analysis. As mentioned above, Co(AFCA)_2_(H_2_O)_4_·H_2_O has the highest catalytic activity on the thermal decomposition of AP, resulting in the fastest burning rate of AP/Co(AFCA)_2_(H_2_O)_4_·H_2_O based solid propellent under the same content. These results indicate that the designed energetic catalysts have good effects on improving the combustion performance of solid propellant.

## Conclusions

In summary, three energetic catalysts, Cu(H_2_O)_4_(AFCA)_2_·H_2_O, Co(H_2_O)_4_(AFCA)_2_·H_2_O, and Fe(H_2_O)_4_(AFCA)_2_·H_2_O, are designed and synthesized based on the coordination reaction of transition metal ions and energetic ligand. Single crystal test indicates that Cu(H_2_O)_4_(AFCA)_2_·H_2_O crystallize in the monoclinic space group, *D*_c_ = 1.918 g cm^−3^. The Co(H_2_O)_4_(AFCA)_2_·H_2_O, and Fe(H_2_O)_4_(AFCA)_2_·H_2_O belong to orthorhombic space group, their density is 1.886 g cm^−3^ and 1.856 g cm^−3^, respectively. In the crystal structure of M(H_2_O)_4_(AFCA)_2_·H_2_O (M = Cu, Co, Fe), each metal ion is hexacoordinated with four water molecules and two AFCA anions, and each furazan-ring presents typical monodentate coordination mode. In addition, the designed catalysts show higher catalytic activity than some reported catalysts such as Co(en)(H_2_BTI)_2_]_2_·en, [Cu_2_(en)_2_ (HBTI)_2_], Co-AzT, Ni-AzT, and [Pb(BTF)(H_2_O)_2_]_*n*_ for the thermal decomposition of AP. When the content of catalyst is 10%, the HTD peak temperatures of AP/Cu(H_2_O)_4_(AFCA)_2_·H_2_O, AP/Co(H_2_O)_4_ (AFCA)_2_·H_2_O, and AP/Fe(H_2_O)_4_(AFCA)_2_·H_2_O are lowered by 120.3 °C, 151.8 °C and 89.5 °C, compared to that of pure AP, and the heat release of which are increased by 768.8 J g^−1^, 780.5 J g^−1^, 750.9 J g^−1^, respectively. In addition, the as-prepared energetic catalysts can also improve the combustion performance of solid propellant. The burning rates of solid propellants composed of AP/Cu(AFCA)_2_(H_2_O)_4_·H_2_O, AP/Co(AFCA)_2_(H_2_O)_4_·H_2_O and AP/Fe(AFCA)_2_(H_2_O)_4_·H_2_O can increased by 2.16 mm s^−1^, 2.53 mm s^−1^, 1.57 mm s^−1^ compared with the case of pure AP. Therefore, the energetic catalysts designed in this study show potential application prospects for improving the combustion performance and energy performance of solid propellants. Meanwhile, this work also provides a reference for the design, preparation and application of other novel energetic catalysts.

## Author contributions

Wei Liu and Yuangang Xu designed the research. Wei Liu carried out experiments and wrote the manuscript. Xiaodong Gou and Fei Xiao performed the morphological and structural characterization. Yulong Zhang and Hanyue Zheng gave suggestions on the experiments and writing. The manuscript was written through contributions of all authors.

## Conflicts of interest

There are no conflicts to declare.

## Supplementary Material

RA-013-D3RA03585A-s001

RA-013-D3RA03585A-s002
