# Supplementary material for: Design, preparation, and combustion performance of energetic catalysts based on transition metal ions (Cu2+, Co2+, Fe2+) and 3-aminofurazan-4-carboxylic acid
Source: RSC Adv. 2023 Sep 5;13(38):26563–73. doi: 10.1039/d3ra03585a (PMC10477948; doi:10.1039/d3ra03585a)
Supplement: RA-013-D3RA03585A-s001 [file RA-013-D3RA03585A-s001.pdf]

Electronic Supplementary Material (ESI) for RSC Advances.
This journal is © The Royal Society of Chemistry 2023

Electronic Supplementary Information

Design, crystal structure, and catalytic mechanism of energetic combustion catalysts based on transition metal ions (Cu^{2+}, Co^{2+}, Fe^{2+}) and 3-aminofurazan-4-carboxylic acid

Wei Liu,*[a] Yuangang Xu,*[b] Yulong Zhang,[c] Hanyue Zheng,[c] Xiaodong Gou[a], and Fei Xiao[a]

[a] School of Environmental and Safety Engineering, North University of China, Taiyuan, Shanxi, China.

[b] School of Chemistry and Chemical Engineering, Nanjing University of Science and Technology, Nanjing, Jiangsu, China.

[c] China North Industry Advanced Technology Generalization Institute, Beijing, China.

* Corresponding authors. E-mail addresses: 1104054142@st.nuc.edu.cn (W. Liu), yuangangxu@163.com (Y. Xu).

**Table of Contents**		
1.	Single-crystal X-ray Diffraction Analysis of $Cu(H_2O)_4(AFCA)_2{\cdot}H_2O$	page 3
2.	Single-crystal X-ray Diffraction Analysis of $Co(H_2O)_4(AFCA)_2{\cdot}H_2O$	page 5
3.	Single-crystal X-ray Diffraction Analysis of $Fe(H_2O)_4(AFCA)_2{\cdot}H_2O$	page 7

1. Single-crystal X-ray Diffraction Analysis of $Cu(H_2O)_4(AFCA)_2 \cdot H_2O$

Table S1. Selected bond lengths [Å] of $Cu(H_2O)_4(AFCA)_2 \cdot H_2O$

parameter	Length (Å)
Cu1-O2W^{i}	1.976 (3)
Cu1-O2W	1.976 (3)
Cu1-O1W	2.313 (3)
Cu1-O1W^{i}	2.313 (3)
Cu1-N1^{i}	2.022 (3)
Cu1-N1	2.022 (3)
O2W-H2WA	0.8674
O2W-H2WB	0.8665
O1W-H1WA	0.8775
O1W-H1WB	0.8774
O3W-H3WA	0.8699
O3W-H3WB	0.8700
O3-N1	1.421 (5)
O3-N2	1.364 (5)
O1-C3	1.262 (5)
O2-C3	1.227 (6)
N1-C1	1.316 (5)
N3-H3A	0.8800
N3-H3B	0.8800
N3-C1	1.313 (6)
N2-C2	1.292 (6)
C1-C2	1.454 (6)
C2-C3	1.505 (6)

Table S2. Selected bond angles [^{o}] for $Cu(H_2O)_4(AFCA)_2 \cdot H_2O$

parameter	bond angel (°)	parameter	bond angel (°)
$O2W^{i}$-Cu1-O2W	180.0	H1WA-O1W-H1WB	109.0
$O2W^{i}$-Cu1-$O1W^{i}$	86.66 (13)	H3WA-O3W-H3WB	109.5
O2W-Cu1-$O1W^{i}$	93.34 (13)	N2-O3-N1	109.7 (3)
$O2W^{i}$-Cu1-O1W	93.34 (13)	O3-N1-Cu1	116.7 (3)
O2W-Cu1-O1W	86.66 (13)	C1-N1-Cu1	137.1 (3)
$O2W^{i}$-Cu1-$N1^{i}$	88.48 (13)	C1-N1-O3	106.2 (3)
$O2W^{i}$-Cu1-N1	91.52 (13)	H3A-N3-H3B	120.0
O2W-Cu1-N1	88.48 (13)	C1-N3-H3A	120.0
O2W-Cu1-$N1^{i}$	91.52 (13)	C1-N3-H3B	120.0
O1W-Cu1-$O1W^{i}$	180.0	C2-N2-O3	107.4 (4)
N1-Cu1-$O1W^{i}$	91.55 (13)	N1-C1-C2	107.1 (4)
$N1^{i}$-Cu1-O1W	91.55 (13)	N3-C1-N1	126.2 (4)
$N1^{i}$-Cu1-$O1W^{i}$	88.45 (13)	N3-C1-C2	126.6 (4)
N1-Cu1-O1W	88.45 (13)	N2-C2-C1	109.7 (4)
$N1^{i}$-Cu1-N1	180.0	N2-C2-C3	123.3 (4)
Cu1-O2W-H2WA	110.7	C1-C2-C3	127.1 (4)
Cu1-O2W-H2WB	110.5	O1-C3-C2	116.3 (4)
H2WA-O2W-H2WB	108.3	O2-C3-O1	127.7 (4)
Cu1-O1W-H1WA	109.8	O2-C3-C2	116.1 (4)
Cu1-O1W-H1WB	110.0		

2. Single-crystal X-ray Diffraction Analysis of $Co(H_2O)_4(AFCA)_2 \cdot H_2O$

Table S3. Selected bond lengths [Å] of $Co(H_2O)_4(AFCA)_2 \cdot H_2O$

parameter	Length (Å)
Co(1)-O(4)#1	2.0769(15)
Co(1)-O(4)	2.0769(15)
Co(1)-O(4)#2	2.0769(15)
Co(1)-O(4)#3	2.0769(15)
Co(1)-N(3)	2.137(2)
Co(1)-N(3)#1	2.137(2)
O(1)-C(1)	1.234(4)
O(2)-C(1)	1.263(4)
O(3)-N(2)	1.369(4)
O(3)-N(3)	1.415(3)
O(4)-H(4A)	0.8691
O(4)-H(4B)	0.8692
N(1)-C(3)	1.338(4)
N(1)-H(1A)	0.8603
N(1)-H(1B)	0.8598
N(2)-C(2)	1.287(4)
N(3)-C(3)	1.315(4)
C(1)-C(2)	1.502(4)
C(2)-C(3)	1.436(4)

Table S4. Selected bond angles [^{o}] for $Co(H_2O)_4(AFCA)_2 \cdot H_2O$

parameter	bond angel (°)	parameter	bond angel (°)
O(4)#1-Co(1)-O(4)	180.00(9)	H(4A)-O(4)-H(4B)	108.2
O(4)#1-Co(1)-O(4)#2	87.32(9)	C(3)-N(1)-H(1A)	120.3
O(4)-Co(1)-O(4)#2	92.68(9)	C(3)-N(1)-H(1B)	119.7
O(4)#1-Co(1)-O(4)#3	92.68(9)	H(1A)-N(1)-H(1B)	120
O(4)-Co(1)-O(4)#3	87.32(9)	C(2)-N(2)-O(3)	107.1(3)
O(4)#2-Co(1)-O(4)#3	180	C(3)-N(3)-O(3)	105.2(2)
O(4)#1-Co(1)-N(3)	91.47(6)	C(3)-N(3)-Co(1)	137.8(2)
O(4)-Co(1)-N(3)	88.53(6)	O(3)-N(3)-Co(1)	117.05(16)
O(4)#2-Co(1)-N(3)	91.47(6)	O(1)-C(1)-O(2)	127.3(3)
O(4)#3-Co(1)-N(3)	88.53(6)	O(1)-C(1)-C(2)	115.6(3)
O(4)#1-Co(1)-N(3)#1	88.53(6)	O(2)-C(1)-C(2)	117.1(3)
O(4)-Co(1)-N(3)#1	91.47(6)	N(2)-C(2)-C(3)	109.6(3)
O(4)#2-Co(1)-N(3)#1	88.53(6)	N(2)-C(2)-C(1)	122.5(3)
O(4)#3-Co(1)-N(3)#1	91.47(6)	C(3)-C(2)-C(1)	127.9(3)
N(3)-Co(1)-N(3)#1	180	N(3)-C(3)-N(1)	125.0(3)
N(2)-O(3)-N(3)	109.9(2)	N(3)-C(3)-C(2)	108.3(2)
Co(1)-O(4)-H(4A)	110.7	N(1)-C(3)-C(2)	126.7(3)
Co(1)-O(4)-H(4B)	110.5	H(5A)-O(5)-H(5B)	108.6

3. Single-crystal X-ray Diffraction Analysis of $Fe(H_2O)_4(AFCA)_2 \cdot H_2O$

Table S5. Selected bond lengths [Å] of $Fe(H_2O)_4(AFCA)_2 \cdot H_2O$

parameter	Length (Å)
$Fe_{(1)}$-$O_{(1W)}$	2.1118(19)
$Fe_{(1)}$-$O_{(1W)}{}^{1}$	2.1118(19)
$Fe_{(1)}$-$O_{(1W)}{}^{2}$	2.1118(19)
$Fe_{(1)}$-$O_{(1W)}{}^{3}$	2.1118(19)
$Fe_{(1)}$-$N_{(1)}$	2.148(3)
$Fe_{(1)}$-$N_{(1)}{}^{3}$	2.148(3)
$O_{(1)}$-$N_{(1)}$	1.428(4)
$O_{(1)}$-$N_{(3)}$	1.369(4)
$O_{(2)}$-$C_{(00C)}$	1.270(4)
$O_{(3)}$-$C_{(00C)}$	1.237(5)
$N_{(1)}$-$C_{(00B)}$	1.315(5)
$N_{(2)}$-$C_{(00B)}$	1.328(5)
$N_{(3)}$-$C_{(00A)}$	1.287(5)
$C_{(00A)}$-$C_{(00B)}$	1.440(5)
$C_{(00A)}$-$C_{(00C)}$	1.504(5)

Table S6. Selected bond angles [^{o}] for $Fe(H_2O)_4(AFCA)_2 \cdot H_2O$

parameter	bond angel (°)	parameter	bond angel (°)
$O_{(1W)}$-$Fe_{(1)}$-$O_{(1W)}^1$	180.0	$N_{(3)}$-$O_{(1)}$-$N_{(1)}$	110.1(3)
$O_{(1W)}^1$-$Fe_{(1)}$-$O_{(1W)}^2$	92.00(11)	$O_{(1)}$-$N_{(1)}$-$Fe_{(1)}$	117.5(2)
$O_{(1W)}$-$Fe_{(1)}$-$O_{(1W)}^2$	88.00(11)	$C_{(00B)}$-$N_{(1)}$-$Fe_{(1)}$	137.7(3)
$O_{(1W)}^1$-$Fe_{(1)}$-$O_{(1W)}^3$	88.00(11)	$C_{(00B)}$-$N_{(1)}$-$O_{(1)}$	104.8(3)
$O_{(1W)}$-$Fe_{(1)}$-$O_{(1W)}^3$	92.00(11)	$C_{(00A)}$-$N_{(3)}$-$O_{(1)}$	106.9(3)
$O_{(1W)}^2$-$Fe_{(1)}$-$O_{(1W)}^3$	180.0	$N_{(3)}$-$C_{(00A)}$-$C_{(00B)}$	109.9(3)
$O_{(1W)}^2$-$Fe_{(1)}$-$N_{(1)}$	91.73(8)	$N_{(3)}$-$C_{(00A)}$-$C_{(00C)}$	122.3(3)
$O_{(1W)}^3$-$Fe_{(1)}$-$N_{(1)}$	88.27(8)	$C_{(00B)}$-$C_{(00A)}$-$C_{(00C)}$	127.9(4)
$O_{(1W)}^2$-$Fe_{(1)}$-$N_{(1)}^1$	88.27(8)	$N_{(1)}$-$C_{(00B)}$-$N_{(2)}$	125.1(3)
$O_{(1W)}^1$-$Fe_{(1)}$-$N_{(1)}^1$	91.73(8)	$N_{(1)}$-$C_{(00B)}$-$C_{(00A)}$	108.4(3)
$O_{(1W)}$-$Fe_{(1)}$-$N_{(1)}$	91.73(8)	$N_{(2)}$-$C_{(00B)}$-$C_{(00A)}$	126.5(4)
$O_{(1W)}^1$-$Fe_{(1)}$-$N_{(1)}$	88.27(8)	$O_{(2)}$-$C_{(00C)}$-$C_{(00A)}$	116.2(4)
$O_{(1W)}^3$-$Fe_{(1)}$-$N_{(1)}^1$	91.73(8)	$O_{(3)}$-$C_{(00C)}$-$O_{(2)}$	127.9(4)
$O_{(1W)}$-$Fe_{(1)}$-$N_{(1)}^1$	88.27(8)	$O_{(3)}$-$C_{(00C)}$-$C_{(00A)}$	115.9(3)
$N_{(1)}$-$Fe_{(1)}$-$N_{(1)}^1$	180.00(3)		
